# Telomeres, Age and Reproduction in a Long-Lived Reptile

**DOI:** 10.1371/journal.pone.0040855

**Published:** 2012-07-13

**Authors:** Virginie Plot, François Criscuolo, Sandrine Zahn, Jean-Yves Georges

**Affiliations:** 1 Université de Strasbourg, Institut Pluridisciplinaire Hubert Curien, Strasbourg, France; 2 CNRS, UMR 7178, Strasbourg, France; Ecole Normale Superieure de Lyon, France

## Abstract

A major interest has recently emerged in understanding how telomere shortening, mechanism triggering cell senescence, is linked to organism ageing and life history traits in wild species. However, the links between telomere length and key history traits such as reproductive performances have received little attention and remain unclear to date. The leatherback turtle *Dermochelys coriacea* is a long-lived species showing rapid growth at early stages of life, one of the highest reproductive outputs observed in vertebrates and a dichotomised reproductive pattern related to migrations lasting 2 or 3 years, supposedly associated with different environmental conditions. Here we tested the prediction of blood telomere shortening with age in this species and investigated the relationship between blood telomere length and reproductive performances in leatherback turtles nesting in French Guiana. We found that blood telomere length did not differ between hatchlings and adults. The absence of blood telomere shortening with age may be related to an early high telomerase activity. This telomere-restoring enzyme was formerly suggested to be involved in preventing early telomere attrition in early fast-growing and long-lived species, including squamate reptiles. We found that within one nesting cycle, adult females having performed shorter migrations prior to the considered nesting season had shorter blood telomeres and lower reproductive output. We propose that shorter blood telomeres may result from higher oxidative stress in individuals breeding more frequently (i.e., higher costs of reproduction) and/or restoring more quickly their body reserves in cooler feeding areas during preceding migration (i.e., higher foraging costs). This first study on telomeres in the giant leatherback turtle suggests that blood telomere length predicts not only survival chances, but also reproductive performances. Telomeres may therefore be a promising new tool to evaluate individual reproductive quality which could be useful in such species of conservation concern.

## Introduction

One of the principal aims of evolutionary ecology is to understand what causes variations in individual fitness. Individuals performing better in terms of survival and reproduction are commonly qualified as “good quality” individuals. This concept is widely used in evolutionary ecology but has rarely been defined in detail and remains difficult to measure [1]. A first step may consist of determining whether individual quality is genetically mediated through natural selection [1], a hypothesis that can be tested using telomere length measurements. Telomeres are repetitive non-coding DNA sequences that cap the ends of eukaryote chromosomes and shorten through successive DNA duplications (i.e. at each cell’s division) until they reach a critical length causing chromosome instability, cell senescence and ultimately cell death [2]. Although telomere length is not necessarily a reliable marker of age, the individual variability in telomere length and telomere shortening has been positively related to longevity or survival across species [3] but also within them [4]. Individual variability of telomere length at a given age is currently explained by the acceleration of telomere loss by stressors (*e.g.* oxidative stress [5]) which may have significant impact during early growth (*e.g.* during accelerated growth [6]–[8]). Telomere loss may however be reduced by telomere maintenance mechanisms such as telomerase activity [9]. Interestingly, although telomere loss with age seems to be the general rule in animals [10], some species are characterized by an unexpected positive relationship between telomere length and age including species such as the Leach’s storm petrel [3], the sand lizard (*Lacerta agilis* [11]) and the water python (*Liasis fuscus* [12]). This leads us to question whether telomere length shortening with age really is a general pattern in the animal kingdom.

The recent use of telomere length as an ageing proxy by ecologists has led to interesting hypotheses concerning sex-specific inheritance and telomere length dynamics in relation to sex-specific life-history strategies [13], [14]. While females have been reported to have longer telomeres than males ([15], [16] but see [17] for a critical review) it has been suggested that telomeres are better predictors of lifetime reproductive success in females than in males, thus favouring selection of longer telomeres in females [14]. Yet the important question of whether telomere dynamics provide any useful information regarding organisms’ fitness related-traits (*e.g.* reproductive success) still needs to be investigated further in different species before being generalized [18].

In light of this context, we investigated telomere length in the leatherback turtle *Dermochelys coriacea*. The leatherback turtle displays many unusual life history traits such as rapid early (and thereafter continuous) growth [19], gigantothermy [20] and high reproductive potential [21] interspersed with pluri-annual inter-breeding oceanic migrations. This makes it an ideal model for (1) testing the prediction of telomere shortening with age *via* the comparison of telomere length in leatherback hatchlings and adult females and (2) investigating the potential relationships between telomere length and individual reproductive strategy.

## Materials and Methods

### Ethics Statement

This project respected the legal requirements of the country in which the work was carried out and followed all institutional guidelines. This study was carried out under CNRS-IPHC institutional license (B67 482 18 delivered by Departmental Direction of the Veterinary Services, Strasbourg, France; and Police Prefecture of Bas-Rhin) and individual licences to JYG (67–220 delivered by the Departmental Direction of the Veterinary Services, Strasbourg, France; and the Police Prefecture of Bas-Rhin; and 973-5/0703039 delivered by the Police Prefecture of French Guiana). Licences covered all field studies and animal experiments, i.e. clinical monitoring of living animals (including biometric measurements and weighing) and sampling on living animals (including blood sampling). Turtles were captured by JYG holder of licenses and VP themselves. All field procedures (except weighing) were performed during oviposition on the natural nesting beach without holding or contention. Biometric measurements and blood sampling were performed once oviposition was initiated for preventing the turtle to abandon its natural nesting behaviour. Biometric and sampling procedures lasted in total 1 minute over the 15 minutes turtles need for laying eggs. Blood samples were collected from the turtles for routine diagnostic purposes (monitoring health, feeding and hormonal states) which include the one specifically concerned by the present study. Weighing occurred after oviposition when the turtle was heading back to the sea. Then, a custom-made harness with four 10 cm wide nylon straps was placed on the sand in front of the turtle, which was secured when the turtle was lying on it. A 4.5 m tall carbon-fibre tripod was immediately placed above the securely harnessed turtle. The turtle was then lifted using a hoist on which an electronic spring scale. Harnessing, lifting, reading the weight, bringing the turtle back down on the sand and releasing the harness lasted in total 5 minutes. Every turtle was individually observed remotely after releasing the harness to ensure she reached safely the sea.

The leatherback turtle is listed as critically endangered at the world scale by the UICN. The area where the fieldwork was performed is part of the Natural Reserve of Amana.

### Field Work

During their reproductive life, sea turtles alternate pluri-annual migrations and reproductive seasons, the latter relying entirely on the use of body reserves stored during the previous migration. During a given reproductive season that may last 2–3 months, females successively lay several clutches during short (2-h) nocturnal landings separated by inter-nesting intervals of ∼10 days at sea [22].

The study site was located at Awala-Yalimapo (5.7°N, 53.9° W), French Guiana, South America, where one of the world’s largest nesting populations of leatherback turtles is located [23]. The study area consisted in a 4-km long stretch of sandy beach that was patrolled every night from 6 pm to 7 am throughout the nesting season (March-July) in 2005 and 2006. There gravid females are individually identified thanks to a long-term tagging program and individually monitored through a pluri-annual capture/mark/recapture (CMR) protocol [23]. This permitted to record the identity and the date of oviposition of every turtle encountered during the considered seasons and to assess the year of their preceding nesting season. Based on similar data, leatherback turtles breeding in French Guiana have been reported to nest mainly every 2 or 3 years [24].

This study concerned 42 adult leatherback females that were regularly observed and captured during the nesting events in 2005 or 2006. For every encounter, individuals’ biometric measurements (Standard Curvilinear Carapace Length, SCCL; Standard Curve Curvilinear Width, SCCW; and body mass) were performed following Georges and Fossette [25]. During the oviposition period, blood samples (∼6 mL) were collected in a heparinised syringe via venipuncture of a venous sinus in the hind flipper. The blood was then transferred to polypropylene microtubes before being placed in a cool box and transported to our field station for centrifugation. Red blood cells and plasma were then separated and placed in Eppendorf tubes and stored at −20°C until laboratory analyses were carried out (see below).

This study focused on adult leatherbacks whose previous reproduction was known thanks to the above-mentioned CMR monitoring already in place on the study site. For these considered individuals, the duration of the migration preceding the nesting season, hereafter referred as RI duration, was estimated by calculating the number of years elapsed between the observed nesting season and the preceding one thanks to the pluri-annual CMR monitoring. This permitted to distinguish individuals with RI of 2 years versus 3 years, as reported by [24], for further comparisons. For example, individuals observed nesting in 2005 were considered as 2-yr and 3-yr remigrants when lastly observed nesting in 2003 and 2002, respectively. The reproductive output during the given nesting season was estimated, and is referred to the Estimated Clutch Frequency (ECF, [26], [27]). The ECF is the total number of clutches that a turtle is believed to have deposited during its nesting season and takes the intermediate unobserved clutches into account (based on the mean inter-nesting duration of 10 days in leatherbacks [27]–[29]) between the first and the last clutch observed.

In addition, in 2009, 20 hatchlings freshly killed by predators were collected, biometric measurements (SCCL, SCCW and body mass) were performed and blood samples were taken. Blood samples were then stored at −20°C until laboratory analyses were carried out.

### Laboratory Analyses

Analyses were carried out at the CNRS-IPHC, Strasbourg, France, following the procedure described by Criscuolo *et al.* [30]. DNA was extracted from 5 µL of red blood cells using a commercial kit (DNeasy Blood and Tissue Kit, QIAGEN). We then performed quantitative Polymerase Chain Reaction (qPCR) assay to measure telomere length, using the method described by Criscuolo *et al.* [30] and adapted to sea turtles. This method [31]determines relative telomere length by measuring within each DNA sample the factor by which it differs from a reference DNA sample in its ratio of telomere repeat copy number (T) to a control gene (C) that was predetermined to be non variable in copy number among the studied population (non-VCN, [32]): i.e. the T/C ratio. This method is widely used for intraspecific comparisons [4], [33]–[35] and has been validated by measurements made on the same samples using qPCR method and telomere restriction fragment method [35]. We tested two control genes: the RNA fingerprint protein 35 (R35) gene (Atlantic leatherback, GenBank: F J039917.1) and the human 18S ribosomal RNA gene. Genes were amplified as follows: R35 gene with primers Turt1 (5′-ATGCTCAGCACCTCACAGG-3′) and Turt2 (5′-TTCATTGATTTCCCGCTAGG-3′), and 18S gene with primers 18S-F (5′-GAGGTGAAATTCTTGGACCGG-3′) and 18S-R (5′-CGAACCTCCGACTTTCGTTCT-3′). We chose to use 18S as the control gene because the amplification efficiency of the qPCR assay and the mean coefficient of intra-individual variation were better (1.02 *vs* 1.06 and 1.8% *vs* 6.46%, respectively). Telomere primers were: Tel1b (5′-CGGTTTGTTTGGGTTTGGGTTTGGGTTTGGGTTTGGGTT-3′) and Tel2b (5′-GGCTTGCCTTACCCTTACCCTTACCCTTACCCTTACCCT-3′). Primers were used at a final isodilution of 100 nM. Telomere and control gene PCR conditions were 10 min at 95°C followed by 30 cycles of 30 s at 56°C, 30 s at 72°C and 60 s at 95°C. Samples (duplicates) were measured using three different plates; inter-plate calibration being carried out via two “golden” samples run as control in each plate. Amplification efficiencies for telomere reached 0.98, 1.05 and 0.99 while those measured for the control gene were 0.98, 1.00 and 1.09. R^2^ of the calibration curves were 0.995, 0.928 and 0.931 for telomere and 0.935, 0.978 and 0.976 for the control gene. Intra-individual variation for telomere Ct values was 3.8±0.40% and values of 1.8% ±0.16% were found for control gene Ct values. Inter-plate variations were 5.6±0.7% for telomere Ct and 3.6±0.5% for control gene Ct (*n* = 20).

### Statistical Analyses

Analyses were conducted using *R* 2.10 (R Development Core Team). Comparisons in blood telomere length between hatchlings and adult females and in blood telomere length between adult females with RI of 2 versus 3 years were assessed using *t*-tests. Models were developed using individual annual reproductive output (ECF) as a variable and biometric measurements at the onset of the breeding season and blood telomere length as factors, including first-degree interactions between factors (Model 1, table 1). Since biometric measurements are supposed to be interdependent, we used a Body Condition Index (BCI) for individuals with complete biometric measurements at the onset of their reproductive season. This BCI corresponded to the residuals of the linear regression of the body mass against a structural index (assessed by the scores of the first PCA axis of the SCCL and SCCW; the proportion of variance explained by the first axis was 83%). Models were then simplified by stepwise deletion of non-significant predictors (α = 0.05), starting with the effect showing the highest p-value [36]. Model selection was based on Akaike’s Information Criterion (AIC). Since the difference in AICs was small (ΔAIC <4) the most parsimonious model was selected. Normal distribution was checked beforehand using the Shapiro-Wilk test. Results are expressed as means ± SE [range].

**Table 1:** Models of the effects of blood telomere length and individual body condition index (BCI) on individual annual reproductive output (ECF) in leatherback turtles nesting in French Guiana in 2005 or 2006.

Model 1 : ECF ∼ BCI + Telomere length + BCI : Telomere length (with 1^st^ degree integration); *df* = 20; *F* = 3.025; *p* = 0.0565; AIC = 97.931				
Model 2 : ECF ∼ BCI + Telomere length; *df* = 20; *F* = 4.759; *p* = 0.021; AIC = 95.979				
	Estimate	Std. error	*t*-value	*p*
Intercept	6.57	0.54	12.185	<0.001
BCI	0.53	0.45	1.185	0.251
Telomere length	0.56	0.20	2.786	0.011
**Model 3 : ECF ∼ Telomere length; ** ***df*** ** = 20; ** ***F*** ** = 7.952; ** ***p*** ** = 0.011; AIC = 95.548**				
	**Estimate**	**Std. error**	***t*** **-value**	***p***
Intercept	6.55	0.54	12.03	<0.001
Telomere length	0.576	0.20	2.82	0.010

## Results

Among the 42 nesting females considered in the present study, 22 individuals were monitored with the complete biometric measurements at the onset of the season. These 22 nesting females were on average 161.0±2.0 [147.5, 179.0] cm long, 116.0±1.0 [108.0, 125.0] cm wide and weighed 429.5±13.3 [304.7, 562.7] Kg, and had laid 7.5±0.5 [2], [11] clutches during their entire nesting season. The 20 hatchlings were 5.66±0.05 [5.00, 6.08] cm long, 4.04±0.04 [3.65, 4.32] cm wide and weighed 41.18±0.51 [35.72, 44.86] g.

There was no significant difference in blood telomere length between hatchlings and adults (T/C ratio: 1.87±0.20 [0.80, 4.32], *n* = 20, *versus* T/C ratios: 1.79±0.28 [0.08, 8.26], *n = *42 respectively*; t* = 0.17, *p* = 0.86).

**Figure 1:**
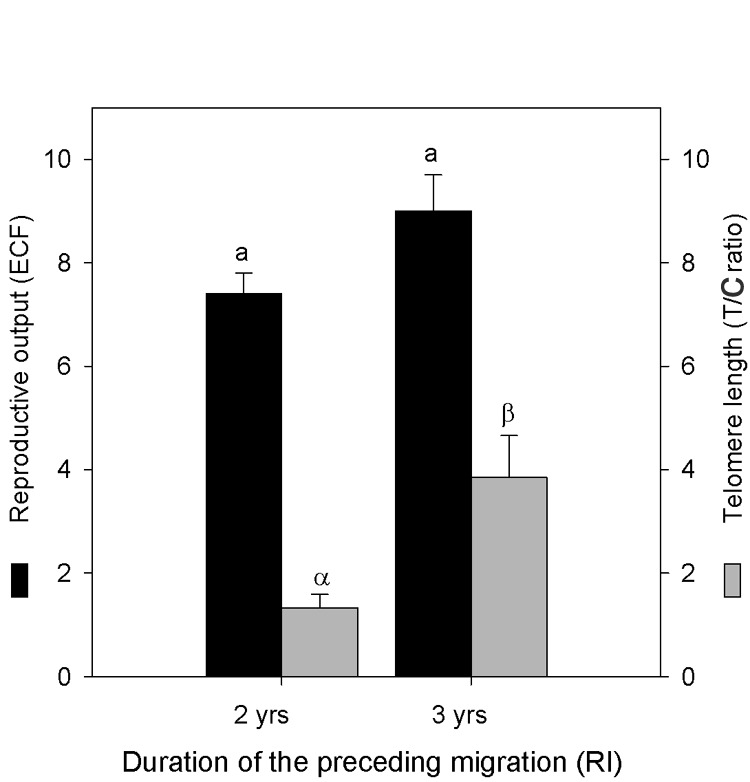
Estimated Clutch Frequency (ECF, the estimated number of clutches laid during the considered nesting season) and blood telomere length in relation to the duration of the migration preceding the considered nesting season (RI, the remigration interval) in leatherback turtles nesting in French Guiana in 2005 or 2006. ECF did not differ according to RI, but tended to be lower for 2-yr RI. Blood telomeres were significantly shorter in females with 2-yr versus 3-yr RI. Letters refer to test significance for ECF and T/C ratio separately. Values are means ± SE.

Among the 42 nesting females considered in the present study, 30 individuals used to be observed during their previous nesting season. The mean RI duration of these 30 individuals was 2.3±0.1 [2], [3] years, most females (21 individuals, i.e. 70%) having a RI of 2 years. Individuals’ annual reproductive output (ECF) did not differ significantly between females with 2-yr versus 3-yr RI (7.4±0.4 [2], [10] clutches, *n* = 21 *versus* 9.0±0.7 [4], [11] clutches, *n* = 9; *t* = −1.98, *p* = 0.057, Fig. 1). Blood telomeres were significantly shorter in females with 2-yr *versus* 3-yr RI (T/C ratios 1.33±0.26 [0.12, 4.75], *n* = 21 *versus* 3.85±0.81 [0.52, 8.26], *n* = 9; *t* = −3.98, *p* = 0.0005, Fig. 1).

Models indicated that female annual reproductive output was better correlated to blood telomere length than to maternal BCI at the onset of the nesting season, and was positively related to blood telomere length (Model 3, Table 1). Similar results were obtained when considering SCCL instead of BCI (initial model ECF ∼ SCCL + T/C + first-degree interaction *F* = 3.503, *p* = 0.037, *AIC* = 96.795; the final model after stepwise backward deletion was identical to Model 3, see Table 1).

## Discussion

Although telomeres have been widely studied as a proxy of cellular and organism senescence in relation to survival and lifespan [37], the potential links between telomere length and reproductive performances have received little attention to date. The present study is the first to investigate the links between telomere length and reproductive performances in sea turtles.

First we found that female reproductive output was better explained by individual blood telomere length than by individual biometrics (body length, body condition index), with the best breeders having longer telomeres. Our findings are consistent with recent studies showing a similar positive relationship between blood telomere length and life reproductive success in birds (dunlins *Calidris alpine* [38]) and sand lizards [14]. This suggests that blood telomere length may be an easily accessible marker of individual reproductive quality in female leatherback turtles. Long term longitudinal monitoring is required, yet difficult to implement, in sea turtles for assessing potential links between telomeres and life reproductive success.

Secondly we show that blood telomeres were shorter in leatherback females breeding after a two-year migration than in those breeding after three years. Female sea turtles restore their body reserves during migration in order to meet the energy requirements for their forthcoming reproduction [22]. On the one hand, telomere dynamic has been reported to be sensitive to oxidative stress [39], which is known to increase concomitantly with reproduction [40]. Our results suggest that the costs of reproduction may be higher in leatherbacks that breed more frequently, but it requires measurement of reproduction-related oxidative stress to be confirmed. Moreover, reproductive output tended to be lower, yet not significantly (*p* = 0.057, see results), in females breeding after 2 years of migration compared to those migrating for 3 years. Similar results were reported in the same population but only in some years [24]. Indeed the “extra-year” used by females migrating 3 years for building their body reserves may be beneficial in terms of reproductive output compared to females migrating for 2 years. In Cyprus nesting site, an inverse relationship was reported in loggerheads (*Caretta caretta*) whereas reproductive effort did not vary with migration duration in green turtles (*Chelonia mydas*, [41]). Therefore, the exact impact of migration duration on reproductive output demands further study.

On the other hand, the dichotomy in migration duration in leatherbacks nesting in French Guiana has been proposed to be related to distinct feeding areas in the Atlantic ocean [42], with individuals feeding in the more southern and coastal areas of West Africa extending their migration by one year compared to individuals feeding in northern oceanic areas [43]. The difference in telomere length reported in the present study may be related to different thermoregulatory constraints during the migration. Indeed higher metabolic costs associated with thermoregulation in northern, cooler, waters during 2-yr migrations may result in higher oxidative stress and ultimately in shorter telomeres. Importantly, individuals may migrate alternatively over 2 or 3 years depending on their nutritional status. Such switching in migration duration may result in complex telomere dynamics throughout an individual lifetime, through varying telomerase activity. Indeed, if telomerase activity reported in somatic tissues of many reptile species [44] holds in sea turtles, delaying intermittently reproduction for one supplementary year may offset previous telomere loss. Long-term longitudinal studies are required to further investigate the potential links between migratory patterns and behaviour, reproductive pattern (breeding every two or three years) and associated reproductive output and hence determine the causes and effects of telomere dynamics on reproductive strategies.

Finally we found no differences in blood telomere length between hatchlings and adults. As the hatchlings considered in our study were individuals predated before reaching the sea, they could be considered poor quality, with shorter telomeres than the hatchlings that do survive. However, the hatchlings used in our study were of a similar weight to successful leatherback hatchlings (44.4±4.16 g, [22]), suggesting this was not the case. Another pitfall when interpreting cross-sectional sampling, which is the case in our study, is that telomere erosion with age may be hidden by the selection of individuals with long telomeres [37]. Yet the longitudinal study of such a relationship in leatherbacks presents technical and logistical constraints: to the best of our knowledge, it is impossible to sample the same individuals from hatchling to adult stages, particularly in the wild. Our results are similar to the unique study published so far on sea turtle telomeres where no correlation was found between blood telomere length and age in captive loggerhead turtles [45]. Yet older individuals tended to have shorter telomeres in epidermis samples [45]. Based on our results and on other studies using telomere length measurements from blood samples, we suggest that the apparent lack of telomere loss with age we found may be attributed to the absence of telomere-based senescence reported in many species of Chelonian [44]. Furthermore, in animal species such as sea turtles where individuals have fast early growth and continuous growth throughout their life, it has been proposed that high telomerase activity (*i.e.* the restoring enzyme of telomeres) occurs in somatic tissues [12]. This process preserves the proliferation capacity of cells during high-rate cell division (generally associated to early growth) through the limitation of telomere loss and can hence prevent senescence [46]. This mechanism has been proposed to be a general characteristic of squamate reptiles [13], [44] and may occur in the leatherback turtle, which is considered to be the fastest-growing turtle [19]. In the sand lizard the fitness of females has been reported to primarily depend on reproductive lifetime and telomere maintenance (*i.e.* telomerase activity) and is therefore subjected to positive selection, unlike males [13]. Such a process, preventing telomeres from early attrition thanks to some telomerase activity, would be beneficial to the survival of leatherback females and may thus represent an adaptive strategy. Assessing telomerase activity in somatic tissues of hatchlings, adult male and female leatherback turtles could be a future step to test this hypothesis. An intriguing issue is also that telomerase activity has been described as inversely related to body mass in rodents, a result interpreted as an anti-tumor protection process [47]. If future studies confirm that telomere length is preserved in adult leatherback turtles (i.e. *a fortiori* through telomerase activity), it would imply a different evolution of the trajectories of control pathways in ageing and cell proliferation by telomeres in testudines.

Monaghan & Haussman [48] stated: “there is a clear need to broaden the range of organisms studied [with respect to telomere dynamics] in order to encompass more variable life histories”. Although more work is needed in the future, telomere studies in sea turtles may represent a useful tool to precisely define which factors act in general on telomere dynamics, and how telomeres relate in turn to life-history decisions, individual survival and reproductive success, which are all fundamental evolutionary questions.
